# To what extent is alcohol consumption in social gatherings associated with observance of COVID-19 restrictions? A rapid review

**DOI:** 10.7189/jogh.12.13001

**Published:** 2022-07-25

**Authors:** Durga Kulkarni, Madhurima Nundy, Emilie McSwiggan, Emily Adams, Marshall Dozier, Karen Hartnup, Amanda Leow, Dudzai Mureyi, Sarah Nelson, Ruth McQuillan, Evropi Theodoratou

**Affiliations:** 1 Usher Network for COVID-19 Evidence Reviews (UNCOVER) group, Usher Institute, University of Edinburgh, Edinburgh, UK; 2 Centre for Global Health, Usher Institute, University of Edinburgh, Edinburgh, UK

## Abstract

**Background:**

Pre-pandemic research found a connection between alcohol consumption and reduced physical distancing among strangers. Understanding the association between alcohol consumption at social gatherings and observance of COVID-19 restrictions can help inform policy related to the safe operation of public spaces where alcohol is typically consumed, as well as guidance related to the safe conduct of social events in private spaces.

**Methods:**

We conducted a rapid review using adapted systematic review methods to explore the association between alcohol consumption in social gatherings and compliance with COVID-19 public health measures and produced a narrative synthesis of our findings. We ran searches in eleven health-related databases (MEDLINE, PubMed, CINAHL, Scopus, Embase (Ovid), ProQuest Public Health, ProQuest Coronavirus, Global Health (Ovid), WHO COVID-19 literature database, PsycInfo (Ovid) and ASSIA) between July 9, 2021, and July 31, 2021. We assessed methodological quality using the relevant Joanna Briggs Institute (JBI) checklists. This review was conducted and reported in accordance with PRISMA-P guidelines.

**Results:**

We identified 7936 studies from the searches. After title, abstract and full-text review, three cross-sectional studies were eligible for inclusion. One study found that people who adhered strongly to physical distancing rules were engaged in about 40% fewer weekly drinks and 60% fewer heavy episodic drinking occasions in a week than people who adhered poorly to physical distancing rules (*P* < 0.01). One study found that people who reported low-risk alcohol consumption patterns had a higher chance of adhering to hand hygiene measures than those who reported high-risk alcohol consumption (odds ratio (OR) = 4.24, 95% confidence interval (CI) = 1.08-16.64). No other statistically significant results on patterns of alcohol consumption and compliance with individual public health measures or with non-pharmaceutical interventions (NPIs) were found. The direction of effect between alcohol consumption and non-adherence to NPIs and the effect of confounding factors has not been established. The quality of studies found was low to moderate, with risk of recall bias and selection bias due to study design; and the extent to which those studies can be generalised beyond their original settings may be limited.

**Conclusions:**

Despite existing evidence suggesting an association between alcohol consumption, reduced physical distancing, and increased social interaction, we found few studies of variable quality exploring the relationship between alcohol consumption and compliance with public health measures. A possible association between higher-risk alcohol behaviours and lower compliance with certain NPIs was suggested, but the direction of effect is unknown, and further studies are required to confirm this finding.

SARS-CoV-2 continues to spread globally with 494 587 638 confirmed cases and 6 170 283 deaths as of April 8, 2022 [1]. The discovery of effective vaccines and new treatments have reduced the proportion of severe disease and deaths in developed countries, but this effort is impeded by emerging variants and remains severe in countries with low overall vaccination rates [2].

Non-pharmaceutical interventions (NPIs), such as physical distancing and hand hygiene necessarily remain at the forefront of COVID-19 mitigation efforts [3,4]. Identifying factors possibly affecting people's compliance with NPI measures must be an ongoing consideration for the effectiveness of public health measures in controlling COVID-19 [5].

Studies carried out before the pandemic identified a connection between alcohol consumption, reduced physical distancing and increased social interaction between strangers [6,7]. As such, understanding the broader picture of the interaction between alcohol and compliance with a range of NPIs is important in guiding policy-making for the safe operation of public spaces. The effect of alcohol consumed at social gatherings (with other people, outside one's own home) is particularly relevant, given increased mixing between households leading to the possibility for broader transmission of COVID-19.

This review aims to summarise and assess the quality of evidence on the links between alcohol consumption at social gatherings and observance of COVID-19 rules such as physical distancing, mask-wearing, and hand washing.

## METHODS

### Protocol

This rapid review was guided by the Preferred Reporting Items for Systematic Review and Meta-Analysis (PRISMA-2020) protocols statement [8]. The study protocol for this rapid review is registered on PROSPERO (CRD42021265206) [9].

### Search strategy

We developed a search strategy by combining three search strings that included terms relating to COVID-19, alcohol, and non-pharmaceutical interventions (NPIs).

We identified initial search terms from indicator papers derived from our scoping searches. We searched English language studies in the following databases: MEDLINE, CINAHL, Embase (Ovid), ProQuest Public Health, Global Health (Ovid), WHO COVID-19 literature database, PsycInfo (Ovid) (seven databases on July 9, 2021), ProQuest Coronavirus, PubMed (both July 13, 2021), Applied Social Sciences Index and Abstracts (ASSIA) (July 14, 2021), and Scopus (July 30, 2021). The draft search strategy was piloted in each database and then finalised.

We also undertook a search in PreVIEW: COVID-19 and reviewed websites offering specific advice with respect to alcohol consumption during the COVID-19 pandemic (July 27, 2021).

The final search strategies used in each database, the results of the PreVIEW: COVID-19 search, and website review are included in Appendix S1 in the **Online Supplementary Document****.**

### Screening and study selection

Deduplication of retrieved records was conducted first in Endnote and then in the Automated Systematic Search Deduplication Tool (ASySD) [10]. ASySD automatically removed clear duplicates and suggested potential duplicates for manual screening and deduplication.

This process was carried out before importing the data set into the systematic review tool, Covidence (COVIDENCE, Melbourne, Australia) [11]. Covidence performed further deduplication in advance of the review team starting work. DK, MN, EA, AL, and SN performed independent double-reviewer screening of titles and abstracts, based on predefined inclusion and exclusion criteria (**Table 1**).

**Table 1:** Criteria for study selection

	Inclusion	Exclusion
**Population**	People consuming alcohol in social gatherings (eg, in bars, pubs, restaurants, parties, house parties)	
	Studies recruiting alcohol non-drinkers, if alcohol non-drinkers constitute the control group	Studies recruiting alcohol non-drinkers, if alcohol non-drinkers do not constitute the control group
**Exposure**	Alcohol consumption/drinking in social gatherings	No alcohol consumption/drinking
**Comparator**	No consumption of alcohol, or consumption of a different amount of alcohol	Single group studies (ie, with no comparator group)
**Outcome**	Observance of COVID-19 rules	COVID-19 outcomes like disease incidence, severity, clinical features, ICU admissions, mortality, etc.
Setting*	Alcohol consumption not explicitly occurring within a single household or alone	Alcohol consumption alone or within a single household
**Study design**	Observational epidemiological studies including cross-sectional and longitudinal studies; Intervention studies like randomised control trials or quasi-experimental studies	Qualitative studies; Systematic reviews, literature reviews; Summaries, viewpoints, newspaper articles and commentaries
**Geographical location**	Studies conducted in any country or countries	(No geographical restrictions applied)
**Language**	Studies published in English	Studies published in languages other than English

*The study was included even if the study setting was unclear or not stated.

Full texts were also independently reviewed by two reviewers. Scoping searches conducted in preparation of this rapid review showed that there would be limited relevant evidence. Therefore, we included studies even if they did not clearly indicate the setting of alcohol consumption.

### Data extraction and management

We piloted our data extraction form on potentially relevant, randomly selected studies identified from our initial scoping searches. We performed data extraction in MS Excel (Microsoft Inc, Seattle WA, USA). For each paper, two reviewers independently extracted data. Any disagreements were resolved by discussion between the team members.

We extracted data on study findings (measures of effect, exposed and control group definition, exposed and control group size, effect estimate, 95% confidence intervals (CIs), and *P*-values) and on study characteristics (first author, publication year, title, country, study design, setting, NPIs addressed, whether data were self-reported, method of data collection, total number of participants, gender, and mean age).

### Quality and risk of bias assessment

Joanna Briggs Institute (JBI) checklists were used for the quality assessment of included studies. In this case, the designs of all three included studies fit best with the Cross-Sectional Studies checklist [12]. We modified the second and fourth items of the checklist to emphasise their relation to the alcohol consumption context (see Appendix S2 in the **Online Supplementary Document**). Independent quality assessment was performed by two reviewers and discrepancies were resolved by discussion. Due to the limited availability of evidence and to ensure maximal comprehensiveness, no studies were excluded on the grounds of poor quality.

### Data synthesis

Owing to the limited availability of evidence and dissimilar measures of effect in different studies, a narrative synthesis of findings was undertaken.

## RESULTS

**Figure 1:**
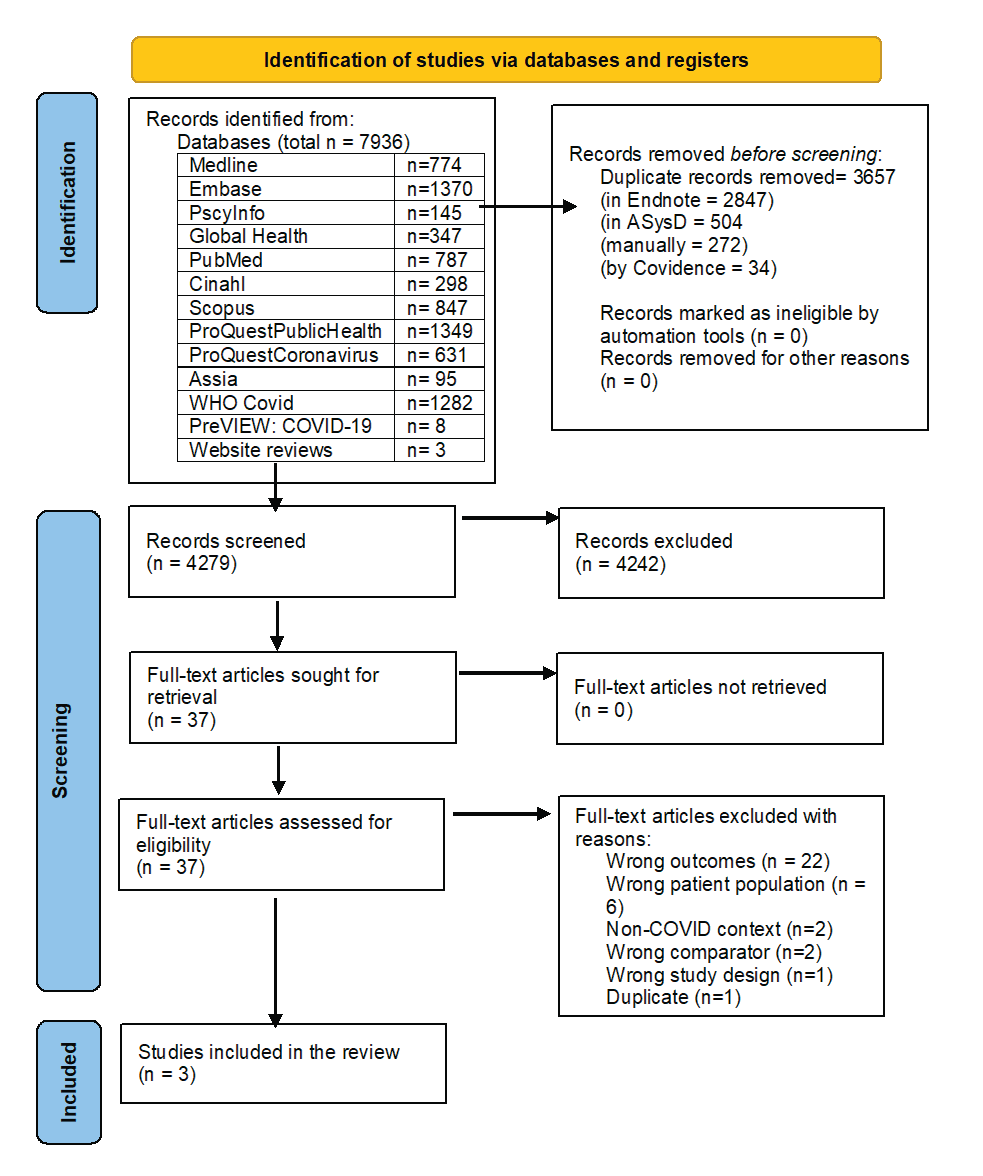
PRISMA diagram showing study identification and selection.

The initial search retrieved 7936 articles. After screening, three studies were eligible for inclusion (**Figure 1**), all of which were cross-sectional studies. The study characteristics are presented in **Table 2**.

**Table 2:** Characteristics of included studies

First author (year)	Country	Context of drinking	Data collection period	NPIs in place during data collection	Data collection method	Method of estimation of alcohol consumption	Sample size	Number of males (%)	Participant age	Covariates adjusted for
Einberger (2021) [13]	USA	Alone; with others online; with others in-person.	April 21, 2020-May 18, 2020	Data were collected during the Washington state-wide “stay home, stay healthy” order that prohibited leaving the house or participating in social gatherings of any kind [14]	Self-reported responses to an online survey.	Daily drinking questionnaire to collect data on the daily number of drinks. Heavy episodic drinking defined as ≥4 drinks for women, ≥5 drinks for men.	560	216 (38.57%)	22 to 28 y (mean = 25.09 y)	Did not adjust for socio-demographic variables; however, the extent of weekly drinking by every individual was accounted for in the analysis of the estimation of the association between drinking in specific contexts (drinking alone, with others virtually, and with others in-person) and NPI compliance.
Hosen (2021) [15]	Bangladesh	Unclear.	April 1, 2020-April 10, 2020	Bangladesh was approximately one month into a two month rapidly executed lockdown that quickly transitioned into general recommendations surrounding distancing and mask-wearing when in public [16].	Self-reported responses to an online survey.	Binary question regarding alcohol drinking (yes/no).	10 067	5650 (56.1%)	≥20 y (skewed younger)	Age, sex, education level, marital status, place/region of residence, occupation, smoking status, and current health status.
Peixoto (2020) [17]	Brazil	Unclear.	May 26, 2020-June 8, 2020	Stay-at-home order in place, barring leaving the house except for essentials. Face masks required outside the home [18]	Self-reported responses to a telephone-based survey.	Low risk: ≤7 (female)≤14 (male) doses/week* High risk >7 (female)>14 (male) doses/week Non-consumption <1 dose/week	5827	2634 (45.2%)	≥50 y	Age, sex, education level, marital status, place/region of residence, skin colour, number of residents in household, and number of self-reported chronic conditions.

*Any drink that contains about 0.6 fluid ounces or 14 g of pure alcohol.

The setting or context of drinking was unspecified in two studies [15,17] so it was unclear if participants consumed alcohol alone, with others in person (eg, at social events), or with others online. Einberger et al. [13] reported whether participants consumed alcohol alone, with others in person or with others online, but data on the exact setting (eg, at home, at social events, restaurants etc.) were not collected.

### Association between alcohol consumption and NPI compliance

The findings of each study are presented in **Table 3** and summarised here.

**Table 3:** Study findings.

First author (year)	Outcome measured	Total number of participants	Definition of the exposed group	Definition of the control group	Number of participants in the exposed group	Number of participants in the control group	Measures of effect	Effect estimate	Upper limit of 95% CI	Lower limit of 95% CI	*P*-value
**Einberger (2021) [** 13 **]**	Physical distancing behaviour in people drinking in isolation.	Unclear.	Self-reported strong adherers.	Self-reported poor adherers.	Unclear.	Unclear.	b (SE)	-0.04 (0.14)	Unclear.	Unclear.	Statistically insignificant (exact value unclear).
	Physical distancing behaviour in people drinking with others online.	Unclear.	Self-reported strong adherers.	Self-reported poor adherers.	Unclear.	Unclear.	b (SE)	0.38 (0.15)	Unclear.	Unclear.	<0.01
	Physical distancing behaviour in people drinking with others in-person.	Unclear.	Self-reported strong adherers.	Self-reported poor adherers.	Unclear.	Unclear.	b (SE)	-0.09 (0.18)	Unclear.	Unclear.	Statistically insignificant (exact value unclear).
	Association of physical distancing behaviour and weekly number of drinks.	Unclear.	Self-reported strong adherers.	Self-reported poor adherers.	Unclear.	Unclear.	Rate ratio calculated by negative binomial regression model	0.61	0.44	0.82	<0.01
	Association of physical distancing behaviour and weekly heavy episodic drinking.	Unclear.	Self-reported strong adherers.	Self-reported poor adherers.	Unclear.	Unclear.	Rate ratio calculated by negative binomial regression model	0.39	0.21	0.72	<0.01
**Hosen (2021) [** 15 **]**	Preventive COVID-19 behaviours.	10 067	Alcohol non-consumers.	Alcohol consumers.	267	9800	b (SE)	1.14 (0.18)	Unclear.	Unclear.	Unclear.
**Peixoto (2020) [** 17 **]**	Physical distancing.	5827	Low- and high-risk alcohol consumers.	Non-consumers of alcohol	1270 in exposed group.	4557 in control group.	OR	High risk = 1.00, low risk = 0.76, control = 1.61	Low risk = 1.56, control = 2.64	Low risk = 0.37, control = 0.98	Unclear.
	Mask wearing.	5827	Low- and high-risk alcohol consumers.	Non-consumers of alcohol	1271 in exposed group.	4558 in control group.	OR	High risk = 1.00, low risk = 0.59, control = 0.94	Low risk = 4.03, control = 1.95	Low risk = 0.09, control = 0.45	Unclear.
	Hand hygiene.	5827	Low- and high-risk alcohol consumers.	Non-consumers of alcohol.	1272 in exposed group.	4559 in control.	OR	High risk = 1.00, low risk = 4.24, control = 1.83	Low risk = 16.64, control = 4.18	Low risk = 1.08, control = 0.8	Unclear.

#### Einberger et al., 2021 [13]

It was observed that people who adhered strongly to physical distancing rules were engaged in about 40% fewer weekly drinks and 60% fewer heavy episodic drinking occasions in a week than people who adhered poorly to physical distancing rules (*P* < 0.01).

People who adhered strongly to physical distancing rules were also more likely to engage in drinking with others online, rather than in person, compared to people who adhered poorly (β = 0.38). Non-significant differences were noted with respect to drinking alone or drinking with others in person.

#### Hosen et al., 2021 [15]

The association between alcohol drinking and preventive COVID-19 behaviours, estimated by linear regression analysis, was observed to be insignificant (β = 1.14, 95% CI = 0.79-1.50). The preventive behaviours analysed together were: frequency of cleaning hands with an alcohol-based rub; mouth- and face-covering while coughing and sneezing; maintaining at least 1m distance from anyone who is coughing or sneezing; and staying home if unwell.

#### Peixoto et al., 2020 [17]

Those who reported low-risk alcohol consumption had a higher chance of adhering to hand hygiene measures than those who reported high-risk alcohol consumption (OR = 4.24, 95% CI = 1.08-16.64). The results were not significant in terms of differences between high-risk, low-risk, and non-consumers of alcohol in adherence to staying-at-home and mask-wearing measures. Precise *P*-values for these analyses were not reported.

### Study quality and risk of bias

Overall study quality varied. The results of our quality assessment are presented in **Table 4**.

**Table 4:** Quality assessment of included studies using modified JBI Quality Appraisal Checklist for cross-sectional studies

	Einberger 2021 [13]	Hosen 2021 [15]	Peixoto 2020 [17]
1. Were the criteria for inclusion in the sample clearly defined?	yes	no	yes
2. Were the study subjects and the setting (in which alcohol consumption occurred) described in detail?	yes	no	no
3. Was the exposure measured in a valid and reliable way?	no	no	no
4. Were objective, standard criteria used for measurement of the condition (alcohol consumption in this case)?	yes	no	yes
5. Were confounding factors identified?	yes	yes	yes
6. Were strategies to deal with confounding factors stated?	yes	yes	yes
7. Were the outcomes measured in a valid and reliable way?	no	no	no
8. Was appropriate statistical analysis used?	no	no	yes
Overall	5/8	2/8	5/8

Some limitations were common across the included studies. Data on alcohol consumption and compliance with NPIs were collected via online surveys in all three studies [13,15,17]. This was unsurprising given the ongoing pandemic, but may raise concerns regarding the validity and reliability of measurement, particularly in contexts such as Bangladesh [15] where the social acceptability of alcohol drinking is low, potentially affecting the reliability of self-reported data.

Another drawback of recruiting participants via online methods is the introduction of selection bias. Selection bias resulting from limited access to internet services may be particularly pertinent to the low- and middle-income country settings of Bangladesh [15] and Brazil [17].

In addition, Hosen et al. [15] estimated compliance with a group of several COVID-19 NPIs combined, rather than examining it separately. This may hide significant differences in compliance with different NPIs at an individual level.

## DISCUSSION

This review was undertaken to analyse and appraise evidence on the association between alcohol drinking in social gatherings and NPI compliance, to guide and inform policies in respect of potential reopening or safe operation of premises serving alcohol, as well as guidance on the safe conduct of social events in private settings, in the COVID-19 pandemic context.

We did not find any studies specifically focused on alcohol consumption in social gatherings. One study [13] examined associations between the strength of young adults’ adherence to physical distancing, and whether they chose to drink with others in person or online. They found that people who adhered strongly to physical distancing rules were more likely to drink with others online than in person. They did not examine the location of in-person drinking (eg, public spaces or venues, friends’ houses), nor the occasions on which such drinking took place (whether special events or everyday socialising). However, their finding suggests that more work might usefully be done to understand the relationship between social drinking and NPI compliance.

Of the three studies included in this review, two found that an increase in the quantity of alcohol consumed was associated with non-compliance to some NPIs: hand hygiene [17] and physical distancing [13]. However, findings from another study [15] and for other NPIs reported in the Peixoto et al. study [17] did not show a significant association between alcohol drinking and NPI compliance.

The strength of association between alcohol consumption in social gatherings and observance of COVID-19 restrictions remains unknown because the included studies did not specify the context in which drinking took place.

### Evidence from the pre-COVID-19 context

Research from before the onset of COVID-19 established a connection between alcohol consumption and the reduction of physical distancing between strangers [6].

Both the physical layout of traditional spaces where alcohol is consumed, such as bars and restaurants, and the psychological effects of alcohol on the brain encourage social interaction with strangers [7,19]. Unfortunately, in the context of an infectious disease outbreak, this can facilitate the transmission of respiratory illnesses [6]. Although the amount of alcohol consumed (discussed below) may be a more significant factor in determining a person’s own behaviour, the setting is important because it determines the risk to others. Risky alcohol consumption in social settings is more likely to affect a broader network of people outside the drinker’s own household than risky consumption at home, with direct implications for the spread of COVID-19.

### Risky health behaviours

Peixoto et al. [17] show that people who meet government-recommended levels for healthy physical activity and people who do not smoke, in addition to those with low-risk alcohol consumption habits, are more likely to report adherence to COVID-19-related NPIs. Hosen et al. [15] also demonstrated that non-smokers and non-drinkers had higher levels of adherence to NPIs.

This maps onto other studies that attempt to identify individual behavioural characteristics as predictors of NPI adherence. Fendrich et al. [20] found that alcohol and drug use consistently served as negative predictors of adherence to physical distancing and personal hygiene measures. Papageorge et al. [21] used nationwide data from the United States to measure adherence to healthy behaviours before and during the COVID-19 pandemic. A significant negative association was found between heart disease and adherence to social distancing, which could be used to argue that those who tend to follow unhealthy lifestyles (if heart disease can be taken as a proxy for such lifestyles) may be expected to engage in fewer self-protective behaviours during a pandemic [21].

Additionally, it was found that very few participants showed a net decline in protective behaviours such as physical distancing or mask-wearing during the pandemic; either no change was recorded or individuals increased adherence significantly [21].

### Time factor

The relationship between time and adherence to NPIs must also be considered. “COVID fatigue” is a colloquial term used to describe feelings of burnout and exhaustion in response to following COVID-19 guidelines. A study from the United States found that every region experienced a reduction in overall rates of adherence to NPIs, from the highest point recorded in early April 2020 to the lowest point in late November 2020, with the greatest difference in social distancing behaviours [22].

As all three studies included in this review were cross-sectional, with data collected between April and June 2020, it is possible that the levels of adherence found were higher than they would be if the studies were conducted later in the pandemic.

### Strengths

This rapid review was conducted in accordance with PRISMA-P guidelines and addressed an important issue that may have implications for SARS-CoV-2 transmission and informing policy. To the best of our knowledge, no other rapid or systematic reviews on this topic have been conducted so far.

### Limitations

This review was restricted to studies in the English language and therefore relevant literature published in other languages may have been excluded.

It is also important to note that governments in different countries imposed different combinations of restrictions at different times during the pandemic. Additionally, all the studies included in this review reported data from the early months of the pandemic. It is likely that population behaviours in response to public health measures will have altered at different stages of the pandemic. This challenges our ability to generalise our findings across different settings.

The overall quality of evidence from this review is very low. Limited existing evidence reflects the uniqueness of our current situation. Mass closures over prolonged periods in many nations are a rare occurrence. The low number of studies found also limited our ability to compare differences based on age, education level, urban or rural setting, or other factors which might show differential drinking behaviours and patterns of compliance with NPIs.

Qualitative studies which explore the social and behavioural aspects, to supplement quantitative research, were excluded from our review. Social drinking is extremely common in some cultures and countries. More research from such contexts is warranted to guide informed reopening policies.

### Directions for future research

The evolution of alcohol-related behaviours during the pandemic and its effects on compliance with public health COVID-19 measures needs to be monitored. Studies based on directly observed (rather than self-reported) behaviour measuring alcohol consumption and NPI compliance in real-time are warranted.

Such evidence will enable public health experts and policymakers to evaluate if there exists a direction of effect and a dose-response relationship between alcohol consumption and NPI compliance. More research on effective public health measures for individuals who are likely to engage in risky behaviours will also help to inform strategies for promoting adherence to pandemic-related safety measures.

### Implications for policy and practice

Limited information on the context in which alcohol consumption took place and the low quality of existing evidence make it difficult to directly link our findings with plans for the safe operation of bars, pubs and restaurants, or with guidance on social gatherings.

## CONCLUSIONS

There is extremely limited evidence on the association between alcohol drinking in social gatherings and NPI compliance during the COVID-19 pandemic.

Our review found an association between alcohol drinking (in non-specified settings) and lower compliance with some NPI measures. The direction of effect between alcohol consumption and non-adherence to NPIs and the effect of confounding factors remain unexplored. The quality of included studies was low to moderate and the extent to which those studies can be generalised to other settings may be limited. Further research in this behavioural field of the COVID-19 pandemic is encouraged.

## Additional material


Online Supplementary Document
