# Supplementary material for: To what extent is alcohol consumption in social gatherings associated with observance of COVID-19 restrictions? A rapid review
Source: J Glob Health. 2022 Jul 25;12:13001. doi: 10.7189/jogh.12.13001 (PMC9308983; doi:10.7189/jogh.12.13001)
Supplement: Online Supplementary Document [file jogh-12-13001-s001.pdf]

Appendix to "To what extent is alcohol consumption in social gatherings associated with observance of COVID-19 restrictions? A rapid review"

S1: LITERATURE SEARCH

Search Dates

Searches were conducted on 9 July 2021 for all databases except PubMed and ProQuest Coronavirus (both 13 July 2021), ASSIA (14 July 2021) and Scopus (30 July 2021). We also undertook a search in PreVIEW: COVID-19 (27 July 2021) and reviewed potential websites (27 July 2021) offering specific advice with respect to alcohol consumption during the COVID-19 pandemic.

Search Strategies

ASSIA (ProQuest)

2021-07-14

Results: 95

(((([STRICT] SU("Alcoholic Beverages" OR "Binge Drinking" OR "Alcohol Drinking" OR "Alcoholic Intoxication")) OR ([STRICT] NOFT(wine* OR beer* OR lager* OR spirits OR whisky OR whiskey)) OR ([STRICT] NOFT(alcohol OR "binge drinking" OR "alcoholic beverage" OR "alcoholic beverages" OR "alcoholic drink" OR "alcoholic drinking" OR "alcoholic drinks")) OR ([STRICT] NOFT(drunk OR drunks OR drunken* OR intoxicat* OR inebriat*))) OR (([STRICT] NOFT(social* OR alone OR house* OR home* OR garden* OR park* OR terrace* OR pub OR pubs OR bar OR bars OR shebeen* OR tavern* OR cafe* OR restaurant* OR hotel* OR hospitality OR venue* OR gig OR gigs OR concert OR concerts OR club OR clubs OR nightclub* OR saloon* OR bistro* OR lounge* OR domestic OR ("waiting room" OR "waiting rooms") OR campus* OR ("student hall" OR "student halls") OR "hall of residence" OR "halls of residence" ("residence hall" OR "residence halls") OR ("student union" OR "student unions") OR party OR parties OR celebrat* OR gathering* OR outside OR inside OR road* OR street* OR festiv* OR football OR sport* OR event OR events OR funeral* OR wake* OR wedding* OR ((("stag night*"))) OR ((("hen night*"))) OR barbeque* OR friend* OR picnic* OR socializ* OR socialis* OR lifestyle)) AND ([STRICT] NOFT(drink*)))) AND (([STRICT] NOFT((covid NEAR/3 complian*) OR restrict* OR mitigation* OR measures OR shielding OR ("face shield" OR "face shields") OR ("face covering" OR "face coverings") OR "N95 respirator*" OR (mask* NEAR/2 wear*) OR (hand NEAR/3 (washing OR wash OR sanitiz* OR sanitis*)) OR hygien* OR distanc* OR "lateral flow" OR NPI* OR "non pharmaceutical intervention*" OR "nonpharmaceutical intervention*" OR "non pharmacological intervention*" OR "nonpharmacological intervention*")) OR ([STRICT] NOFT(contact NEAR/3 tracing)) OR ([STRICT] NOFT(track NEAR/2 trace)) OR ([STRICT] NOFT("check in" OR "check out")) OR ([STRICT] NOFT((infect* OR disease*) NEAR/2 (prevent* OR control*))) OR ([STRICT] SU("Communicable Disease Control" OR "Contact Tracing" OR "Physical Distancing" OR "Quarantine" OR "Masks" OR "N95

Respirators" OR "Ventilation")) OR ([STRICT] NOFT("stay at home" OR lockdown* OR quarantin* OR confinement* OR isolat* OR ventilation)))) AND (([STRICT] SU(Coronavirus OR Betacoronavirus OR Coronavirus Infections OR COVID-19 OR SARS-CoV-2)) OR (STRICT NOFT("Wuhan seafood market pneumonia virus")) OR (STRICT NOFT(2019nCoV OR Betacoronavirus* OR Corona Virus* OR Coronavirus* OR Coronovirus* OR CoV OR CoV2 OR COVID OR COVID19* OR COVID-19* OR COVID-2019* OR HCoV-19 OR nCoV OR SARS2 OR SARSCoV OR SARS-CoV OR 2019-nCoV-19 OR SARS-CoV-2 OR SARS CoV 2 OR SARS-CoV-2019)))

CINAHL

2020-07-09

Results: 298

Unqualified search for free text, searching the following fields: Title, Abstract, Subject headings, PubMed ID (PMID), Digital Object Identifier (DOI). Author

S21	S18 and S19 and S20
S20	S1 or S2 or S3 or S4 or S5 or S6 or S7 or S8 or S9 or S10
S19	S11 or S12 or S13 or S14 or S15 or S16 or S17
S18	((MH "Coronavirus Infections") or (MH "Coronavirus") or (MH "COVID-19") or (MH "SARS-CoV-2")) or ("Wuhan seafood market pneumonia virus" or 2019nCoV or Betacoronavirus* or "Corona Virus*" or Coronavirus* or Coronovirus* or CoV or CoV2 or COVID or COVID19* or "COVID-19*" or "COVID-2019*" or "HCoV-19" or nCoV or SARS2 or SARSCoV or "SARS-CoV" or "2019-nCoV-19" or "SARS-CoV-2” or "SARS CoV 2" or "SARS-CoV-2019")
S17	(MH "Alcohol Drinking+")
S16	(MH "Alcoholic Beverages+")
S15	"wine* or beer* or lager* or spirits or whiskey or whisky"
S14	(MH "Alcoholic Intoxication")
S13	alcohol or "binge drinking" or "alcoholic beverage*" or "alcoholic drink*"
S12	drunk or drunks or drunken* or intoxicat* or inebriat*
S11	((drink*) and (social* or alone or house* or home* or garden* or park* or terrace* or pub or pubs or bar or bars or shebeen* or tavern* or cafe* or restaurant* or hotel* or

hospitality or venue* or gig or gigs or concert or concerts or club or clubs or nightclub* or saloon* or bistro* or lounge* or domestic or "waiting room*" or campus* or "student hall*" or "hall of residence" or "halls of residence" or "residence hall*" or "student union*" or party or parties or celebrat* or gathering* or outside or inside or road* or street* or festiv* or football or sport* or event or events or funeral* or wake* or wedding* or "stag night*" or "hen night*" or barbeque* or friend* or picnic* or socializ* or socialis* or lifestyle)

(MH "Handwashing") or (MH "Contact Tracing") or (MH "Infection Control")

(MH "Social Distancing") or (MH "Stay-at-Home Orders") or (MH "Quarantine")

S10

(MH "Masks") or (MH "N95 Respirators") or (MH "Respiratory Protective Devices")

S9

S8

S7 shielding or "face-shield*" or "mask-wearing" or "face-covering*" or (mask* N2 wear*) or "N95 respirator*"

S6 (covid N3 complian*) or (restrict* or mitigation* or measures or (hand N3 (washing or wash or sanitiz* or sanitis*)) or hygien* or distanc* or lateral flow or NPI* or "nonpharmaceutical intervention*" or "non-pharmaceutical intervention*" or "non-pharmacological intervention" or "nonpharmacological intervention"

S5 (contact N3 tracing) or (track N2 trace) or "check-in" or "check-out"

S4 (infect* or disease*) N2 (prevent* or control*)

S3 "stay-at-home" or lockdown* or confinement* or quarantin* or isolat* or "self-isolat*"

S2 (MH "Ventilation")

S1 ventilation

[Text Wrapping Break]

Coronavirus (ProQuest)

2021-07-13

Results: 631

((([STRICT] SU("Alcoholic Beverages" OR "Binge Drinking" OR "Alcohol Drinking" OR "Alcoholic Intoxication")) OR ([STRICT] NOFT(wine* OR beer* OR lager* OR spirits OR whisky OR whiskey)) OR ([STRICT] NOFT(alcohol OR "binge drinking" OR "alcoholic beverage" OR "alcoholic beverages" OR "alcoholic drink" OR "alcoholic drinking" OR "alcoholic drinks")) OR ([STRICT] NOFT(drunk OR drunks OR drunken* OR intoxicat* OR inebriat*))) OR (([STRICT] NOFT(social* OR alone OR house* OR home* OR garden* OR park* OR terrace* OR pub OR pubs OR bar OR bars OR shebeen* OR tavern* OR cafe* OR restaurant* OR hotel* OR hospitality OR venue* OR gig OR gigs OR concert OR concerts OR club OR clubs OR nightclub* OR saloon* OR bistro* OR lounge* OR domestic OR ("waiting room" OR "waiting rooms") OR campus* OR ("student hall" OR "student halls") OR "hall of residence" OR “halls of residence” OR ("residence hall" OR "residence halls") OR ("student union" OR "student unions") OR party OR parties OR celebrat* OR gathering* OR outside OR inside OR road* OR street* OR festiv* OR football OR sport* OR event OR events OR funeral* OR wake* OR wedding* OR ((("stag night*"))) OR ((("hen night*"))) OR barbeque* OR friend* OR picnic* OR socializ* OR socialis* OR lifestyle)) AND ([STRICT] NOFT(drink*)))) AND (([STRICT] NOFT((covid NEAR/3 complian*) OR restrict* OR mitigation* OR measures OR shielding OR ("face shield" OR "face shields") OR ("face covering" OR "face coverings") OR "N95 respirator*" OR (mask* NEAR/2 wear*) OR (hand NEAR/3 (washing OR wash OR sanitiz* OR sanitis*)) OR hygien* OR distanc* OR "lateral flow" OR NPI* OR "non pharmaceutical intervention*" OR "nonpharmaceutical intervention*" OR "non pharmacological intervention*" OR "nonpharmacological intervention*")) OR ([STRICT] NOFT(contact NEAR/3 tracing)) OR ([STRICT] NOFT(track NEAR/2 trace)) OR ([STRICT] NOFT("check in" OR "check out")) OR ([STRICT] NOFT((infect* OR disease*) NEAR/2 (prevent* OR control*))) OR ([STRICT] SU("Communicable Disease Control" OR "Contact Tracing" OR "Physical Distancing” OR "Quarantine" OR "Masks" OR "N95 Respirators" OR "Ventilation")) OR ([STRICT] NOFT("stay at home" OR lockdown* OR quarantin* OR confinement* OR isolat* OR ventilation)))

Embase (Ovid)

Embase <1980 to 2021 Week 26>

2021-07-09

Results: 1370

1 drinking behavior/

2 exp alcoholic beverage/

3 (wine* or beer* or lager* or whiskey or whisky or spirits).mp.

4 binge drinking/ or exp alcohol intoxication/ or exp alcohol consumption/

5 (alcohol or binge drinking or alcoholic beverage* or alcoholic drink*).mp.

6 (drunk or drunks* or drunken* or intoxicat* or inebriat*).mp.

7 (drink* and (social* or alone or house* or home* or garden* or park* or terrace* or pub or pubs or bar or bars or shebeen* or tavern* or cafe* or restaurant* or hotel* or hospitality or venue* or gig or gigs or concert or concerts or club or clubs or nightclub* or saloon* or bistro* or lounge* or domestic or waiting room* or campus* or student hall* or "hall* of residence" or residence hall* or student union* or party or parties or celebrat* or gathering* or outside or inside or road* or street* or festiv* or football or sport* or event or events or funeral* or wake* or wedding* or stag night* or hen night* or barbeque* or friend* or picnic* or socializ* or socialis* or lifestyle)).mp.

8 Coronavirus Infection/ or Coronavirus Disease 2019/ or Coronavirinae/ or Betacoronavirus/ or exp Severe acute respiratory syndrome coronavirus 2/

9 Wuhan seafood market pneumonia virus

10 (2019nCoV or Betacoronavirus* or Corona Virus* or Coronavirus* or Coronovirus* or CoV or CoV2 or COVID or COVID19* or COVID-19* or COVID-2019* or HCoV-19 or nCoV or SARS2 or SARSCoV or SARS-CoV or 2019-nCoV-19 or SARS-CoV-2 or SARS CoV 2 or SARS-CoV-2019).mp.

11 communicable disease control/ or exp quarantine/ or social distancing/

12 exp mask/

13 (shielding or face shield* or face covering* or n95 respirator* or (mask* adj2 wear*)).mp.

14 ((covid adj3 complian*) or (restrict* or mitigation* or measures) or (hand adj3 (washing or wash or sanitiz* or sanitis*)) or hygien* or distanc* or lateral flow or NPI* or non pharmaceutical intervention* or nonpharmaceutical intervention* or nonpharmacological intervention* or non pharmacological intervention*).mp.

15 ((contact adj3 tracing) or (track adj2 trace) or check in or check out).mp.

16 ((infect* or disease*) adj2 (prevent* or control*)).mp.

17 (stay-at-home or lockdown* or quarantin* or confinement* or isolat*).mp.

18 ventilation.mp.

19 or/1-7

20 8 or 9 or 10

21 or/11-18

22 19 and 20 and 21

Global Health (Ovid)

Results: 2021-07-09

Results: 347

1 alcohol intake/

2 exp alcoholic beverages/

3 (wine* or beer* or lager* or whisky or whiskey or spirits).mp.

4 (alcohol or binge drinking or alcoholic beverage* or alcoholic drink*).mp.

5 (drunk or drunks* or drunken* or intoxicat* or inebriat*).mp.

6 public houses/

7 (drink* and (social* or alone or house* or home* or garden* or park* or terrace* or pub or pubs or bar or bars or shebeen* or tavern* or cafe* or restaurant* or hotel* or hospitality or venue* or gig or gigs or concert or concerts or club or clubs or nightclub* or saloon* or bistro* or lounge* or domestic or waiting room* or campus* or student hall* or "hall* of residence" or residence hall* or student union* or party or parties or celebrat* or gathering* or outside or inside or road* or street* or festiv* or football or sport* or event or events or funeral* or wake* or wedding* or stag night* or hen night* or barbeque* or friend* or picnic* or socializ* or socialis* or lifestyle)).mp.

8 Betacoronavirus/ or Human Coronaviruses/ or exp severe acute respiratory syndrome-related coronavirus/

9 (Wuhan seafood market pneumonia virus).mp.

10 (2019nCoV or Betacoronavirus* or Corona Virus* or Coronavirus* or Coronovirus* or CoV or CoV2 or COVID or COVID19* or COVID-19* or COVID-2019* or HCoV-19 or nCoV or SARS2 or SARSCoV or SARS-CoV or 2019-nCoV-19 or SARS-CoV-2 or SARS CoV 2 or SARS-CoV-2019).mp.

11 masks/

12 (shielding or face shield* or face covering* or n95 respirator* or (mask* adj2 wear*)).mp.

13 ((covid adj 3 complian*) or (restrict* or mitigation* or measures) or (hand adj3 (washing or wash or sanitiz* or sanitis*)) or hygien* or distanc* or lateral flow or NPI* or non

pharmaceutical intervention* or nonpharmaceutical intervention* or non pharmacological intervention* or nonpharmacological intervention*).mp.

14 ((contact adj3 tracing) or (track adj2 trace) or check in or check out).mp.

15 infection control/ or contact tracing/ or quarantine/

16 ((infect* or disease*) adj2 (prevent* or control*)).mp.

17 (stay-at-home or lockdown* or quarantin* or confinement* or isolat*).mp.

18 ventilation/ or natural ventilation/ or ventilation.mp.

19 or/1-7

20 8 or 9 or 10

21 or/11-18

22 19 and 20 and 21

Page Break

MEDLINE (Ovid)

Ovid MEDLINE(R) and Epub Ahead of Print, In-Process, In-Data-Review & Other Non-Indexed Citations, Daily and Versions(R) <1946 to July 08, 2021>

2021-07-09

Results: 774

1 exp Alcohol Drinking/

2 exp Alcoholic Beverages/

3 (wine* or beer* or lager* or spirits or whisky or whiskey).mp.

4 alcoholic intoxication/ or binge drinking/

5 (alcohol or binge drinking or alcoholic beverage* or alcoholic drink*).mp.

6 (drunk or drunks* or drunken* or intoxicat* or inebriat*).mp.

7 (drink* and (social* or alone or house* or home* or garden* or park* or terrace* or pub or pubs or bar or bars or shebeen* or tavern* or cafe* or restaurant* or hotel* or hospitality or venue* or gig or gigs or concert or concerts or club or clubs or nightclub* or saloon* or bistro* or lounge* or domestic or waiting room* or campus* or student hall* or "hall* of residence" or residence hall* or student union* or party or parties or celebrat* or gathering* or outside or inside or road* or street* or festiv* or football or sport* or event

or events or funeral* or wake* or wedding* or stag night* or hen night* or barbeque* or friend* or picnic* or socializ* or socialis* or lifestyle)).mp.

8 Coronavirus/ or Betacoronavirus/ or Coronavirus Infections/ or COVID-19/ or SARS-CoV-2/

9 Wuhan seafood market pneumonia virus.mp.

10 (2019nCoV or Betacoronavirus* or Corona Virus* or Coronavirus* or Coronovirus* or CoV or CoV2 or COVID or COVID19* or COVID-19* or COVID-2019* or HCoV-19 or nCoV or SARS2 or SARSCoV or SARS-CoV or 2019-nCoV-19 or SARS-CoV-2 or SARS CoV 2 or SARS-CoV-2019).mp.

11 communicable disease control/ or contact tracing/ or physical distancing/ or quarantine/

12 masks/ or n95 respirators/

13 (shielding or face shield* or face covering* or n95 respirator* or (mask* adj2 wear*)).mp.

14 ((covid adj 3 complian*) or (restrict* or mitigation* or measures) or (hand adj3 (washing or wash or sanitiz* or sanitis*)) or hygien* or distanc* or lateral flow or NPI* or non pharmaceutical intervention* or nonpharmaceutical intervention* or non pharmacological intervention* or nonpharmacological intervention*).mp.

15 ((contact adj3 tracing) or (track adj2 trace) or check in or check out).mp.

16 ((infect* or disease*) adj2 (prevent* or control*)).mp.

17 (stay-at-home or lockdown* or quarantin* or confinement* or isolat*).mp.

18 Ventilation/ or ventilation.mp.

19 or/1-7

20 or/8-10

21 or/11-18

22 19 and 20 and 21

PsycInfo (Ovid)

APA PsycInfo <1806 to July Week 1 2021>

2021-07-09

Results: 145

1 drinking behavior/

2 exp alcohol drinking patterns/

3 exp alcoholic beverages/

4 (wine* or beer* or lager* or spirits or whiskey or whisky).mp.

5 alcohol intoxication/

6 (alcohol or binge drinking or alcoholic beverage* or alcoholic drink*).mp.

7 (drunk or drunks or drunken* or intoxicat* or inebriat*).mp.

8 (drink* and (social or alone or house* or home* or garden* or park* or terrace* or pub or pubs or bar or bars or shebeen* or tavern* or cafe* or restaurant* or hotel* or hospitality or venue* or gig or gigs or concert or concerts or club or clubs or nightclub* or saloon* or bistro* or lounge* or domestic or waiting room* or campus* or student hall* or "hall* of residence" or residence hall* or student union* or party or parties or celebrat* or gathering* or outside or inside or road* or street* or festiv* or football or sport* or event or events or funeral* or wake* or wedding* or stag night* or hen night* or barbeque* or friend* or picnic* or socializ* or socialis* or lifestyle)).mp.

9 coronavirus/

10 (Wuhan seafood market pneumonia virus).mp.

11 (2019nCoV or Betacoronavirus* or Corona Virus* or Coronavirus* or Coronovirus* or CoV or CoV2 or COVID or COVID19* or COVID-19* or COVID-2019* or HCoV-19 or nCoV or SARS2 or SARSCoV or SARS-CoV or 2019-nCoV-19 or SARS-CoV-2 or SARS CoV 2 or SARS-CoV-2019).mp.

12 exp Public Health/

13 personal protective equipment/

14 preventive health behavior/ or prevention/ or quarantine/

15 disease transmission/

16 (shielding or face shield* or face covering* or n95 respirator* or (mask* adj2 wear*)).mp.

17 ((covid adj 3 complian*) or (restrict* or mitigation* or measures) or (hand adj3 (washing or wash or sanitiz* or sanitis*)) or hygien* or distanc* or lateral flow or NPI* or non pharmaceutical intervention* or nonpharmaceutical intervention* or non pharmacological intervention* or nonpharmacological intervention*).mp.

18 ((contact adj3 tracing) or (track adj2 trace) or check in or check out).mp.

19 ((infect* or disease*) adj2 (prevent* or control*)).mp.

20 (stay-at-home or lockdown* or quarantin* or confinement* or isolat*).mp.

21 ventilation.mp.

22 or/1-8

23 9 or 10 or 11

24 or/12-21

25 22 and 23 and 24

Public Health (ProQuest)

2021-07-09

Results: 1349

Proquest Public Health Database

(MESH("Alcohol Drinking" or "Alcoholic Beverages" or "Alcoholic Intoxication" or "Binge Drinking") or TIAB(wine* or beer* or lager* or spirits or whisky or whiskey) or TIAB(drunk or drunks* or drunken* or intoxicat* or inebriat*) or TIAB(alcohol or “binge drinking” or "alcoholic beverage*" or "alcoholic drink*") or (drink* and TIAB(social* or alone or house* or home* or garden* or park* or terrace* or pub or pubs or bar or bars or shebeen* or tavern* or cafe* or restaurant* or hotel* or hospitality or venue* or gig or gigs or concert or concerts or club or clubs or nightclub* or saloon* or bistro* or lounge* or domestic or "waiting room*" or campus* or "student hall" or “student halls” or "hall of residence" or “halls of residence” or "residence hall" or “residence halls” or "student union*" or party or parties or celebrat* or gathering* or outside or inside or road* or street* or festiv* or football or sport* or event or events or funeral* or wake* or wedding* or "stag night*" or "hen night*" or barbeque* or friend* or picnic* or socializ* or socialis* or lifestyle))) AND (MESH("Coronavirus Infections" or "Coronavirus" or “Betacoronavirus ” or “Covid-19” or “SARS-COV2”) or ("Wuhan seafood market pneumonia virus" or 2019nCoV or Betacoronavirus* or "Corona Virus*" or Coronavirus* or Coronovirus* or CoV or CoV2 or COVID or COVID19* or COVID-19* or COVID-2019* or HCoV-19 or nCoV or SARS2 or SARSCoV or SARS-CoV or 2019-nCoV-19 or SARS-CoV-2 or “SARS CoV 2” or SARS-CoV-2019)) AND (TIAB(covid n/3 complian*) or (restrict* or mitigation* or measures or “face covering*” or “face shield*” or “N95 respirator*” or (mask* n/2 wear*) or (hand n/3 (washing or wash or sanitiz* or sanitis*)) or hygien* or distanc* or “lateral flow” or NPI* or "non pharmaceutical intervention*" or "nonpharmaceutical intervention*" or "non pharmacological intervention*" or "nonpharmacological intervention*") or TIAB((contact n/3 tracing) or (track n/2 trace) or "check in" or "check out") or ((infect* or disease*) n/2 (prevent* or control*)) or MESH("Contact Tracing" or "Quarantine" or "Communicable

Disease Control") or ("stay at home" or lockdown* or quarantin* or isolat* or confinement*) or “ventilation” or MESH("Ventilation" or "Masks"))

PubMed

2020-07-13

Results: 787

20 #7 and #10 and #19

19 #11 or #12 or #13 or #14 or #15 or #16 or #17 or #18

18 (Ventilation[MeSH]) or ventilation[Text Word]

17 "stay at home"[Text Word] or lockdown*[Text Word] or quarantin*[Text Word] or confinement*[Text Word] or isolat*[Text Word] or “self isolat*”[Text Word]

16 “Infection prevention”[Text word] or “infection control”[Text word] or “disease prevention”[Text word] or “disease control”[Text word]

15 “contact tracing”[Text Word] or “track and trace”[Text Word] or "check in"[Text Word] or "check out"[Text Word]

14 ((covid[Text Word] AND complian*[Text Word]) or (restrict*[Text Word] or mitigation*[Text Word] or measures[Text Word]) or (hand[Text Word] AND wash[Text Word]) or handwashing[Text Word] or (hand[Text Word] AND sanitiz*[Text Word]) or (hand[Text Word] AND sanitis*[Text Word]) or hygien*[Text Word] or distanc*[Text Word] or "lateral flow"[Text Word] or NPI[Text Word] or NPIs[Text Word] or “non pharmaceutical intervention*”[Text Word] or “nonpharmaceutical intervention*”[Text Word] or “non pharmacological intervention*”[Text Word] or “nonpharmacological intervention*”[Text Word])

13 shielding[Text Word] or “face shield*”[Text Word] or “face covering*”[Text Word]) or “n95 respirator*”[Text Word] or (mask[Text Word] AND wear[Text Word]) or (mask[Text Word] AND wearing[Text Word]) or (masks[Text Word] AND wear[Text Word]) or (masks[Text Word] AND wearing[Text Word])

12 (("masks"[MeSH) or (n95 respirators[MeSH]))

11 ((("communicable disease control"[MeSH]) or ("contact tracing"[MeSH])) or ("physical distancing"[MeSH])) or ("quarantine"[MeSH])

10 #8 or #9

9 "Wuhan seafood market pneumonia virus"[Text Word] or 2019nCoV[Text Word] or Betacoronavirus*[Text Word] or "Corona Virus*"[Text Word] or Coronavirus*[Text Word] or Coronovirus*[Text Word] or CoV[Text Word] or CoV2[Text Word] or COVID[Text Word] or COVID19*[Text Word] or COVID-19*[Text Word] or COVID-2019*[Text Word] or HCoV-19[Text Word] or nCoV[Text Word] or SARS2[Text Word] or SARSCoV[Text Word] or SARS-CoV[Text Word] or 2019-nCoV-19[Text Word] or SARS-CoV-2[Text Word] or “SARS CoV 2”[Text Word] or SARS-CoV-2019[Text Word]

8 ((("coronavirus"[MeSH]) or ("Betacoronavirus"[MeSH]) or ("coronavirus infections"[MeSH])) or ("covid 19"[MeSH])) or ("sars cov 2"[MeSH])

7 #1 or #2 or #3 or #4 or #5 or #6

6 drink*[Text Word] and (social*[Text Word] or alone[Text Word] or house*[Text Word] or home*[Text Word] or garden*[Text Word] or park*[Text Word] or terrace*[Text Word] or pub[Text Word] or pubs[Text Word] or bar[Text Word] or bars [Text Word] or shebeen* [Text Word] or tavern*[Text Word] or cafe*[Text Word] or restaurant*[Text Word] or hotel*[Text Word] or hospitality[Text Word] or venue*[Text Word] or gig[Text Word] or gigs[Text Word] or concert[Text Word] or concerts[Text Word] or club[Text Word] or clubs[Text Word] or nightclub*[Text Word] or saloon*[Text Word] or bistro*[Text Word] or lounge*[Text Word] or domestic[Text Word] or "waiting room*"[Text Word] or campus*[Text Word] or "student hall*"[Text Word] or "hall of residence"[Text Word] or "halls of residence"[Text Word] or "residence hall*"[Text Word] or "student union*"[Text Word] or party[Text Word] or parties[Text Word] or celebrat*[Text Word] or gathering*[Text Word] or outside[Text Word] or inside[Text Word] or road*[Text Word] or street*[Text Word] or festiv*[Text Word] or football[Text Word] or sport*[Text Word] or event[Text Word] or events[Text Word] or funeral*[Text Word] or wake*[Text Word] or wedding*[Text Word] or "stag night*"[Text Word] or "hen night*"[Text Word] or barbeque*[Text Word] or friend[Text Word] or picnic*[Text Word] or socializ*[Text Word] or socialis*[Text Word] or lifestyle[Text Word])

5 drunk[Text Word] or drunks*[Text Word] or drunken*[Text Word] or intoxicat*[Text Word] or inebriat*[Text Word]

4 alcohol[Text Word] or “binge drinking”[Text Word] or “alcoholic beverage*”[Text Word] or “alcoholic drink*[Text Word]

3 ("alcoholic intoxication"[MeSH])) or (“binge drinking”[MeSH])

2 wine*[Text Word] or beer*[Text Word] or lager*[Text Word] or spirits[Text Word] or whisky[Text Word] or whiskey[Text Word]

1 ((("alcohol drinking"[Mesh]) or ("alcoholic beverages"[MeSH]))

Scopus

2021-07-30

Results: 847

(TITLE-ABS-KEY ("Wuhan seafood market pneumonia virus" OR 2019ncov OR betacoronavirus* OR "Corona Virus*" OR coronavirus* OR coronovirus* OR cov OR cov2 OR covid OR covid19* OR covid-19* OR covid-2019* OR hcov-19 OR ncov OR sars2 OR sarscov OR sars-cov OR 2019-ncov-19 OR sars-cov-2 OR "sars cov 2" OR sars-cov-2019)) AND ((TITLE-ABS-KEY (wine* OR beer* OR lager* OR spirits OR whisky OR whiskey OR alcohol OR “binge drinking” OR “alcoholic beverage*” OR “alcoholic drink” OR drunk OR drunks* OR drunken* OR intoxicat* OR inebriat*)) OR ((TITLE-ABS-KEY (drink*)) AND (TITLE-ABS-KEY (social* OR alone OR house* OR home* OR garden* OR park* OR terrace* OR pub OR pubs OR bar OR bars OR shebeen* OR tavern* OR cafe* OR restaurant* OR hotel* OR hospitality OR venue* OR gig OR gigs OR concert OR concerts OR club OR clubs OR nightclub* OR saloon* OR bistro* OR lounge* OR domestic OR "waiting room*" OR campus* OR "student hall*" OR "hall* of residence" OR "residence hall*" OR "student union*" OR party OR parties OR celebrat* OR gathering* OR outside OR inside OR road* OR street* OR festiv* OR football OR sport* OR event OR events OR funeral* OR wake* OR wedding* OR "stag night*" OR "hen night*" OR barbeque* OR friend* OR picnic* OR socializ* OR socialis* OR lifestyle)))) AND ((TITLE-ABS-KEY (covid W/3 complian*)) OR (TITLE-ABS-KEY (shielding OR face shield*" OR "face covering*" OR “N95 respirator*” OR restrict* OR mitigation* OR measures OR hygien* OR distanc* OR "lateral flow" OR npi* OR "non pharmaceutical intervention*" OR "nonpharmaceutical intervention*" OR "non pharmacological intervention*" OR "nonpharmacological intervention*" OR "stay-at-home" OR lockdown* OR quarantin* OR confinement* OR isolat* OR "check in" OR "check out" OR ventilation)) OR (TITLE-ABS-KEY (mask* W/2 wear*)) OR (TITLE-ABS-KEY (hand W/3 (washing OR wash OR sanitiz* OR sanitis*))) OR (TITLE-ABS-KEY ((contact W/3 tracing) OR (track W/2 trace))) OR (TITLE-ABS-KEY ((infect* OR disease*) W/2 (prevent* OR control*))))

WHO COVID-19 literature database

2021-07-09

Results: 1282

1. (tw:(wine*)) OR (tw:(beer*)) OR (tw:(lager*)) OR (tw:(whiskey)) OR (tw:(whisky)) OR (tw:(spirits)) OR (tw:(Alcohol)) OR (tw:("binge drinking")) OR (tw:(drunk)) OR (tw:(drunks)) OR (tw:(drunken*)) OR (tw:(intoxicat*)) OR (tw:(inebriat*)) OR (tw:("Alcoholic drink")) OR (tw:("alcoholic drinks")) OR (tw:("alcoholic beverage")) OR (tw:("alcoholic beverages"))

2. ((tw:((tw:(drink*)))) AND ((tw:(tw:(social*)) OR (tw:(alone*)) OR (tw:(house*)) OR (tw:(home*)) OR (tw:(garden*)) OR (tw:(park*)) OR (tw:(terrace*)) OR (tw:(pub)) OR (tw:(pubs)) OR (tw:(bar)) OR (tw:(bars)) OR (tw:(shebeen*)) OR (tw:(tavern*)) OR (tw:(cafe*)) OR (tw:(restaurant*)) OR (tw:(hotel*)) OR (tw:(hospitality)) OR (tw:(venue*)) OR (tw:(gig)) OR (tw:(gigs)) OR (tw:(concert)) OR (tw:(concerts)) OR (tw:(club)) OR (tw:(clubs)) OR (tw:(nightclub*)) OR (tw:(saloon*)) OR (tw:(bistro*)) OR (tw:(lounge*)) OR (tw:(domestic)) OR (tw:("waiting room")) OR (tw:("waiting rooms")) OR (tw:(campus*)) OR (tw:("student hall")) OR (tw:("student halls")) OR (tw:("hall of residence")) OR (tw:("halls of residence")) OR (tw:("residence hall")) OR (tw:("residence halls")) OR (tw:("student union")) OR (tw:("student unions")) OR (tw:(party)) OR (tw:(parties)) OR (tw:(celebrat*)) OR (tw:(gathering*)) OR (tw:(outside)) OR (tw:(inside)) OR (tw:(road*)) OR (tw:(street*)) OR (tw:(festiv*)) OR (tw:(football)) OR (tw:(sport*)) OR (tw:(event)) OR (tw:(events)) OR (tw:(funeral*)) OR (tw:(wake*)) OR (tw:(wedding*)) OR (tw:("hen night")) OR (tw:("hen nights")) OR (tw:("stag night") OR (tw:("stag nights")) OR (tw:(barbeque*)) OR (tw:(friend*)) OR (tw:(picnic*)) OR (tw:(socializ*)) OR (tw:(socialis*)) OR (tw:(lifestyle)))))

3. (tw:(shielding)) OR (tw:("face shield")) OR (tw:("face shields") OR (tw:("face covering")) OR (tw:("face coverings")) OR (tw:("face-covering")) OR (tw:("face-coverings")) OR (tw:("face mask")) OR (tw:("face masks")) OR (tw:("face-mask")) OR (tw:("face-masks")) OR (tw:("N95 respirator")) OR (tw:("N95 respirators"))) OR (tw:(restrict*)) OR (tw:(measures)) OR (tw:(mitigation*)) OR (tw:(handwashing)) OR (tw:("hand-washing")) OR (tw:("hand washing")) OR (tw:(handsanit*)) OR (tw:(hand sanitiser)) OR (tw:("hand sanitizer")) OR (tw:(hygien*)) OR (tw:(distanc*)) OR (tw:(npi*)) OR (tw:("non-pharmaceutical intervention")) OR (tw:("non-pharmaceutical interventions")) OR (tw:("nonpharmaceutical intervention")) OR (tw:("nonpharmaceutical interventions")) OR (tw:("non

pharmaceutical intervention")) OR (tw:("non pharmaceutical interventions")) OR (tw:("non-pharmacological intervention")) OR (tw:("non-pharmacological interventions")) OR (tw:("nonpharmacological intervention")) OR (tw:("nonpharmacological interventions")) OR (tw:("non pharmacological intervention")) OR (tw:("non pharmacological interventions")) OR (tw:("contact tracing") OR (tw:("track and trace")) OR (tw:("check-in")) OR (tw:("check in")) OR (tw:("check out")) OR (tw:("check-out")) OR (tw:(stay-at-home)) OR (tw:(lockdown*)) OR (tw:(quarantin*)) OR (tw:(confinement)) OR (tw:(isolat*)) OR (tw:("self-isolating")) OR (tw:(selfisolating)) OR (tw:(ventilation))

4. 1 OR
5. 3 and 4

PreVIEW:COVID-19 results and website reviews

**Database / Website / Source**	**Search terms / Keywords (include date of search)**	**Number of relevant results**	**References & Links**
preVIEW : COVID-19	(title) COVID-19 AND (title/abstract) ALCOHOL (26/07/2021)	8	□ Global COVID-19 spread: socioeconomic determinants and lessons for future pandemics<br>Authors: Nicholas Ngepah<br>10.21203/rs.3.rs-31764/v1
			□ Knowledge, beliefs, mental health, substance use, and behaviors related to the COVID-19 pandemic among U.S. adults: A national online survey<br>Authors: Ralph J. DiClemente, Ariadna Capasso, Shahmir H. Ali, Abbey M. Jones, Joshua Foreman, Yesim Tozan<br>10.21203/rs.3.rs-44532/v1
			□Factors associated with drinking behaviour during COVID-19 social distancing and lockdown among adults in the UK<br>Authors: Claire Garnett, Sarah E Jackson, Melissa Oldham, Jamie Brown, Andrew Steptoe, Daisy Fancourt<br>10.1101/2020.09.22.20199430

			□ COVID-19 among bartenders and waiters before and after pub lockdown Authors: Fredrik Methi, Kjetil Telle, Karin Magnusson<br>10.1101/2021.02.01.21250905
			Alcoholism as a Risk Factor for COVID-19: Boosting Inflammatory Response<br>Authors: Muhammad Akram, Mehwish Iqbal, Marcos Altable, Juan Moises de la Serna<br>10.20944/preprints202103.0547.v1
			Higher Alcohol Consumption Is Not Linked To Higher SARS CoVid19 Infection and Death<br>Authors: Yugal Kishore Mohanta, Abeer Hashem, Elsayed Fathi Abd_Allah, Tapan Kumar Mohanta<br>10.21203/rs.3.rs-30073/v1
			Host genetic liability for severe COVID-19 overlaps with alcohol drinking behavior and diabetic outcomes and in over 1 million participants<br>Authors: Frank R Wendt, Antonella De Lillo, Gita A Pathak, Flavio De Angelis, - COVID-19 Host Genetics Initiative, Renato Polimanti<br>10.1101/2020.11.08.20227884
			□ A global survey on changes in the supply, price and use of illicit drugs and alcohol, and related complications during the 2020 COVID-19 pandemic<br>Authors: Ali Farhoudian, Seyed Ramin Radfar, Hossein Mohaddes Ardabili, Parnian Rafei, Mohsen Ebrahimy, Arash Khojasteh Zonoozi, Cornelis A J De Jong, Mehrnoosh Vahidi, Masud Yunesian, Christos Kouimtsidis, Shalini Arunogiri, Helena Hansen, Kathleen T Brady, Marc N Potenza, - ISAM-PPIG Global Survey Consortium, Alexander Mario Baldacchino, Hamed Ekhtiari<br>10.1101/2020.07.16.20155341
The King's Fund	COVID-19 topic search	0	

The Joseph Rowntree Foundation	COVID-19 topic search	0	
IRISS	COVID-19 & alcohol	0	
Alcohol Health Alliance	COVID-19 topic search	0	
Alcohol Change UK	COVID-19 topic search	0	
Public Health Scotland Website<br><br>www.publichealthscotland.scot	COVID019 & alcohol	0	
Public Health England Website https://www.gov.uk/government/organisations/public-health-england	COVID-19 alcohol	0	
Scottish Government www.gov.scot	COVID-19 alcohol	0	
World Health Organisation	COVID-19 alcohol	1	Brief report on key information relating to alcohol consumption and COVID-19, including comment that "alcohol alters your thoughts, judgement, decision-making and behaviour". ,https://www.euro.who.int/__dat

www.who.int			a/assets/pdf_file/0010/437608/Alcohol-and-COVID-19-what-you-need-to-know.pdf
World Health Organisation Europe www.euro.who.int	COVID-19 alcohol	1	FAQ document outlining key questions raised in relation to alcohol and COVID-19; however, no specific mention of impact of alcohol consumption on NPI adherence. https://www.euro.who.int/en/health-topics/disease-prevention/alcohol-use/publications/2020/fact-sheet-alcohol-and-covid-19-what-you-need-to-know-2020/frequently-asked-questions-faq-about-alcohol-and-covid-19
Centers for Disease Control www.cdc.gov	COVID-19 alcohol	1	Link to WHO document above entitled “What you need to know”.

S2: JOANNA BRIGGS INSTITUTE CHECKLIST FOR CROSS-SECTIONAL STUDIES (MODIFIED)

We assessed methodological quality using the Joanna Briggs Institute checklist for Cross-Sectional Studies (Joanna Briggs Institute, 2020) with minor amendments to address the relationship to our study question. The amendments we made to the original checklist are shown in bold below:

1. Were the criteria for inclusion in the sample clearly defined?
2. Were the study subjects and the setting **(in which alcohol consumption occurred)** described in detail?
3. Was the exposure measured in a valid and reliable way?
4. Were objective, standard criteria used for measurement of the condition **(alcohol consumption in this case)**?
5. Were confounding factors identified?
6. Were strategies to deal with confounding factors stated?
7. Were the outcomes measured in a valid and reliable way?
8. Was appropriate statistical analysis used?
